# A Gift from Vacation: New Rash on His Foot

**DOI:** 10.5811/cpcem.2016.11.31125

**Published:** 2017-01-18

**Authors:** Rachel A. Lindor, Christopher S. Russi

**Affiliations:** Mayo Clinic, Department of Emergency Medicine, Rochester, Minnesota

A 32-year-old man sought care at the emergency department for evaluation of a rash on his foot, three weeks after returning from a beach vacation in the Caribbean. He reported that the rash had appeared one week earlier, was severely pruritic, and seemed to be expanding daily. He reported no systemic symptoms and had normal vital signs. Examination demonstrated two raised, erythematous, serpiginous lesions on the plantar aspect of his left foot (Imaage).

Cutaneous larva migrans (CLM) is an infection caused by several different types of hookworm. Infected animal hosts, usually cats and dogs, shed hookworm eggs in their feces. When CLM develops from animal sources it is often referred to as *hookworm-related CLM*.^1^ The eggs hatch into larvae that can survive for several weeks under the right conditions – most commonly warm sand or soil in tropical or subtropical areas, including the Gulf Coast states. Larvae are most often transmitted to people walking barefoot through these areas, although hands and buttocks are also common sites of entry. The larvae release degradative enzymes to penetrate through the epidermis, which causes the characteristic rash several days to several weeks after exposure. The track may extend several millimeters per day as the larvae migrate through the skin.^2^ Hookworm may cause systemic infections in animal hosts, but most species lack the collagenase necessary to penetrate the dermis in humans and are therefore confined to the skin.^3^

CLM is diagnosed clinically on the basis of the classic finding of a pruritic, serpiginous rash in the setting of recent travel to an endemic area. Although the infection is self-limited because the larvae die within five to six weeks, treatment is often required to address the severe pruritus. Oral antihelminthics (albendazole or ivermectin) are effective, possible therapeutic options.^1^

## Figures and Tables

**Image:**
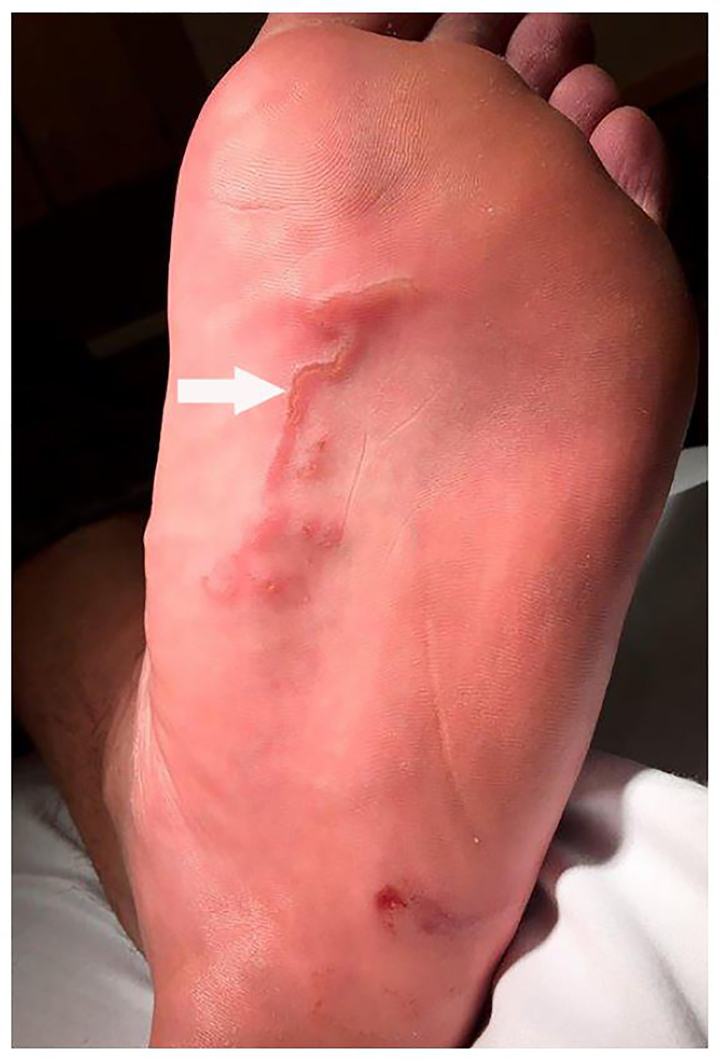
Rash associated with cutaneous larva migran (arrow). Erythema on the heel is not part of the rash.
